# Parameter-free rendering of single-molecule localization microscopy data for parameter-free resolution estimation

**DOI:** 10.1038/s42003-021-02086-1

**Published:** 2021-05-11

**Authors:** Adrien C. Descloux, Kristin S. Grußmayer, Aleksandra Radenovic

**Affiliations:** 1 École Polytechnique Fédérale de Lausanne, Laboratory of Nanoscale Biology, Lausanne, Switzerland; 2 Present Address: Delft University of Technology, Grußmayer Lab, Department of Bionanoscience, Kavli Institute of Nanoscience, Delft, The Netherlands

**Keywords:** Optical imaging, Image processing

## Abstract

Localization microscopy is a super-resolution imaging technique that relies on the spatial and temporal separation of blinking fluorescent emitters. These blinking events can be individually localized with a precision significantly smaller than the classical diffraction limit. This sub-diffraction localization precision is theoretically bounded by the number of photons emitted per molecule and by the sensor noise. These parameters can be estimated from the raw images. Alternatively, the resolution can be estimated from a rendered image of the localizations. Here, we show how the rendering of localization datasets can influence the resolution estimation based on decorrelation analysis. We demonstrate that a modified histogram rendering, termed bilinear histogram, circumvents the biases introduced by Gaussian or standard histogram rendering. We propose a parameter-free processing pipeline and show that the resolution estimation becomes a function of the localization density and the localization precision, on both simulated and state-of-the-art experimental datasets.

## Abstract (web summary)

Descloux et al. introduce a parameter-free modified histogram rendering method for resolution estimation of localization microscopy datasets compatible with decorrelation analysis. The proposed bilinear histogram rendering and processing pipeline convey the localization information into the image accurately, making the resolution estimate dependent on the localization precision and the localization density.

## Introduction

In 2019 we proposed a novel method to estimate resolution using decorrelation analysis on a single image^1^. A partial phase autocorrelation for a series of filtered images determines the highest spatial frequency with sufficiently high signal to noise ratio. The method has now been tested for more than a year across imaging modalities using the open source software (https://github.com/Ades91/ImDecorr). We received overall positive comments from specialists ranging from two-photon microscopy to structured illumination imaging and beyond. Initially, we presented decorrelation analysis on the single-molecule localization microscopy symposium (SMLMS), looking for feedback from this community that relies heavily on image processing. Unlike other super-resolution methods, most SMLM software does not directly output an image, but a set of localizations that need to be rendered for visualization. This adds another level of complexity to the interpretation of the images. Interactive discussions around increasing localization precision in optimized SMLM (due to new developments in experiments^2–4^ and SMLM software^5^) and the resulting demands on localization density prompted us to investigate in greater detail the best practice of using our algorithm.

Ideally, the estimated resolution should not change when choosing a different method to render the dataset. Nevertheless, commonly used rendering choices (fixed and localization-uncertainty-based Gaussian rendering) are implying additional assumptions about the underlying localization statistics, which can impact the resolution estimate^6^. In our publication, we rendered all the localizations as a Gaussian with a standard deviation equal to their respective localization uncertainty. The estimation of the localization uncertainty is a non-trivial task and depends on many parameters such as camera calibration or the noise model used^7,8^. In addition, there exist localization softwares using e.g., machine learning that do not routinely return localization estimates^9^. In our recent addendum^6^, we advise, based on simulations, to choose histogram rendering to construct input images for decorrelation analysis. We show that the use of fixed Gaussian rendering is not optimal, as it is likely to bias the resolution estimate and does not provide any benefits compared to histogram rendering. We also discussed the challenges of using histogram rendering in conjunction with our resolution estimation algorithm.

Here, we focus on diverse experimental data, pinpointing the limits of SMLM and decorrelation analysis and proposing a workflow for accurate resolution estimation. We present a modified histogram method for SMLM dataset rendering that is compatible with resolution estimation using decorrelation analysis. The proposed bilinear histogram rendering method does not require the knowledge of the localization uncertainty estimate or artificial jittering of the localizations and minimizes the rounding error of naïve histogram rendering. We demonstrate that the modified histogram rendering is able to convey the localization information into the image accurately. Using experimental data and simulations, we show how our resolution estimate depends on the rendering pixel size and localization density. We find that our method expects a localization density of about 1–4×10^4^ loc. per µm^2^ to work reliably with experimental data.

## Results

### Histogram and bilinear histogram rendering

**Fig. 1:**
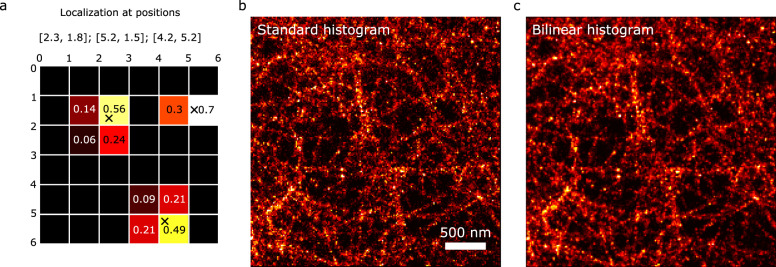
Illustration of bilinear rendering of localization microscopy data. **a** Schematic of the rendering of localizations with positions [2.3, 1.8], [5.2, 1.5], [4.2, 5.2] (black crosses) on the image grid, with the weights indicated. **b** Standard histogram rendering applied on the center region (pixel size of 15 nm; FOV of 3×3 µm) of the cSir Phalloidin dataset (see Fig. 2 for details). **c** Bilinear histogram rendering of the same region as in **b**. Scale bar 500 nm.

**Fig. 2:**
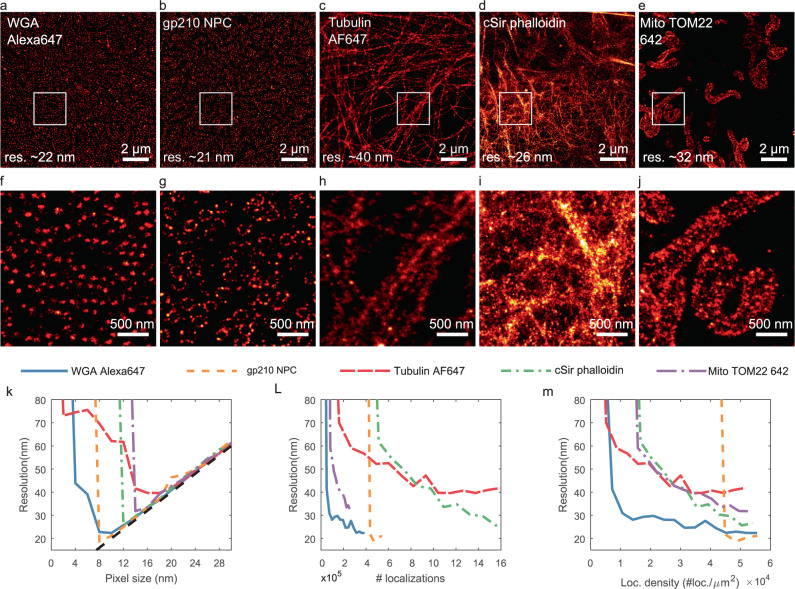
Experimental resolution estimate using bilinear histogram rendering. **a**–**e** Selection of Shareloc.xyz data rendered with a pixel size of 5 nm (field-of-view of 12 × 12 µm^2^), scale bar: 2 µm. The resolution indicated corresponds to the smallest resolution estimated from all the tested pixel sizes. **f**–**j** Zoom-in of **a**–**e** of 2.5 × 2.5 µm^2^ regions indicated by the white squares, scale bar: 500 nm. **k** Resolution vs pixel size. **l** Resolution as a function of number of localizations. **m** Resolution as a function of localization density.

Let us consider an SMLM dataset of N localizations with positions $\left[{x}_{n},{y}_{n}\right]$. One way to render the data without making additional assumptions is to plot them as a histogram, which is usually expressed as

1 $$I\left[x,y\right]={\sum }_{n}^{N}\delta \left[x-\left\lfloor {x}_{n}\right\rfloor ,y-\left\lfloor {y}_{n}\right\rfloor \right]$$

where $\left\lfloor {x}_{n}\right\rfloor$ denotes the $x$ position of the ${n}^{{th}}$ localization event floored to the nearest integer multiple of the chosen pixel size. This rounding operation is problematic as it introduces a round-off error, which can be detrimental to the resolution estimation, especially if the pixel size is on the order of the localization uncertainty. In order to alleviate this effect, we propose to render the data using a modified histogram expression

2 $$I\left[x,y\right]={\sum }_{n}^{N}\delta \left[x-\left\lfloor {x}_{n}\right\rfloor ,y-\left\lfloor {y}_{n}\right\rfloor \right]* {w}_{n}\left[x,y\right]$$

where $*$ denotes the convolution operation of a discrete Dirac distribution with the 2 × 2 matrix ${w}_{n}$. Compared to Eq. 1, each localization is spread on the four nearest pixels and the weights are given by

3 $${w}_{n}\left[{x}_{n},{y}_{y}\right]=\left(\begin{array}{c}1-{y}_{n}+\left\lfloor {y}_{n}\right\rfloor \\ {y}_{n}-\left\lfloor {y}_{n}\right\rfloor \end{array}\right)\otimes \left(\begin{array}{cc}1-{x}_{n}+\left\lfloor {x}_{n}\right\rfloor , & {x}_{n}-\left\lfloor {x}_{n}\right\rfloor \end{array}\right)$$

where $\otimes$ denotes the tensor product. This rendering approach is equivalent to a linear (or bilinear for the 2D case) interpolation and is also equivalent to a 2D average shifted histogram with an infinitesimal shift^10^. The weight distribution of three localizations is illustrated in Fig. 1a. Figure 1b and c show the rendering of the same dataset (central region of cSir phalloidin dataset presented in Fig. 2) using standard histogram rendering (b) and bilinear histogram rendering (c). We see that the standard histogram image looks more pixelated (due to the rounding operation) while the bilinear histogram looks noticeably smoother.

This rendering method will be referred to as bilinear histogram rendering. We note that this rendering approach is not new^11^ but it is the first time, to our knowledge, that it is used in the context of resolution estimation. With this approach, the information about the position of the localization is conveyed into the image and it is, to our knowledge, the most appropriate rendering choice for resolution estimation based on decorrelation analysis^1^. The resolution estimation assumes that the image is expressed as the sum of a signal (exhibiting spatial correlations) and uncorrelated noise. The estimation relies on the computation of cross-correlation coefficients ${d}$ between the high-passed version of the input image and its low-pass filtered Fourier normalized version. Normalizing the Fourier transform balances the contributions of signal and noise. The low-pass filter consists in an ideal lowpass filter characterized by its cutoff frequency $r$, which allows to compute the cross-correlation coefficient as a function of $r$. With decreasing ***r***, first noise contributions are gradually removed while preserving the bandwidth-limited signal. The asymmetry (noise rejection but signal preservation) is mathematically translated into an increase in the correlation. Eventually, the low-pass filter will start to remove the signal which reduces the correlation. The presence of a local maximum in the function $d\left(r,\sigma \right)$ indicates the spatial frequency of best noise rejection and signal preservation. The high-pass filter is an inverted Gaussian shaped filter of standard deviation $\sigma$. We have demonstrated that the image resolution can be estimated from the function $d\left(r,\sigma \right)$ by finding, for each value of ${{\sigma }}_{i}$, the position ${r}_{i}$ of the local maxima. The image cutoff is then defined as the largest ${r}_{i}$.

In the case of SMLM data, the resulting resolution estimate depends on the density of localizations, the homogeneity of the labeling, the filtering of high uncertainty localizations and the accuracy of the drift correction. A Matlab implementation of the bilinear histogram method used in this work is made publicly available at https://github.com/Ades91/ImDecorr [10.5281/zenodo.4655984].

**Fig. 3:**
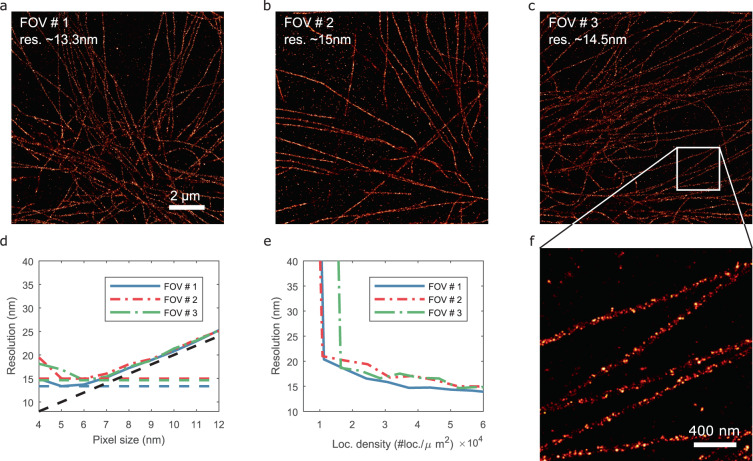
Resolution estimation of DNA-PAINT data. **a–c** Bilinear histogram of microtubule data at 5 nm pixel size, scale bar: 2 µm. **d** Resolution as a function of the pixel size. **e** Resolution as a function of the loc. density. **f** Zoom in of **c**, scale bar: 400 nm.

The only remaining open parameter for rendering is the pixel size. If it is set too small, the localizations might not exhibit any spatial correlations as gaps will appear between localizations, which is likely to result in an underestimated resolution. If the pixel size is set too large, our resolution estimation will possibly be close to twice the pixel size, which means that the Nyquist sampling criterion is not fulfilled. Similarly, if the localization density is too low, such that no continuous structures can form, the algorithm will also underestimate the resolution (see Supplementary Information, Note and Figs. 1, 2 and 3 for a comparison between standard and bilinear histogram and Gaussian rendering).

### Experimental results

In order to demonstrate the applicability and advantages of the proposed bilinear histogram rendering method for resolution estimation of SMLM, we applied the discussed methodology on several SMLM datasets, including (d)STORM, DNA-PAINT and multi-color STORM. Although most journals mandate or encourage data sharing, comprehensive and up-to-date database(s) of localization microscopy are still rare. In our opinion, they are an invaluable asset for tool developers and the SMLM community.

### Experimental results: ShareLoc

We first applied our method on several single channel (d)STORM datasets publicly available at https://shareloc.xyz/. We selected five different proteins from different cellular structures acquired by three different groups (see Table 1 below for details).

**Table 1:** Selected https://shareloc.xyz/#/ data.

Name	Description	Author	Hash	Loc. Prec.
1 WGA A647.smlm	dSTORM of central canal of Xenopus nuclear pore complex	orestis.faklaris@mri.cnrs.fr	22d97bd241f21f27b1bee29666a622c5	~8 nm
tubulin-AF647_4.smlm	–	Markus Sauer, University of Wuerzburg	2ef45b60988e370cf80dfe994524245b	–
COS7_csir_phalloidin.smlm	actin stained with caged Si-rhodamine phalloidin of a Cos-7 cell	Markus Sauer, University of Wuerzburg, DOI: 10.1002anie.201509649	41080d3e899715bed8ecee58192138c8	~6 nm
gp210 NPC.smlm	dSTORM of Xenopus nuclear pore complex	orestis.faklaris@mri.cnrs.fr	5e56796222dca3bceba7a6d6e3b64e5d	~6 nm
s1-c2-fixed-tom22-642-30ms_1_mmstack_pos0.smlm	Mitochondria image used for figure 6b	wei.ouyang@pasteur.fr	94106847488a2e145b5e062ed0addbb2	–

Figure 2a–e shows the five selected data sets rendered at their highest localization density using bilinear histogram rendering with a pixel size of 5 nm. The resolution indicated corresponds to the smallest resolution estimated from all the tested pixel size. For comparison purposes, a field-of-view of 12 × 12 µm^2^ is chosen for all data sets. Figure 2f–j shows a 2.5 × 2.5 µm^2^ zoom-in of Fig. 2a–e, indicated by the white squares. Figure 2k shows the resolution estimate as a function of the pixel size at their highest localization density. We see that for large pixel size, the resolution evolves linearly at twice the pixel size (sampling limited). As the pixel size decreases, the resolution estimate reaches a minimum depending on the data set and then rises again (sparsity limited). The minimum of each curve corresponds to the resolution indicated in Fig. 2a–e. Figure 2l shows the estimated resolution as a function of the number of localizations included in the analysis. For each sample point, we computed the resolution as a function of the pixel size and retained only the smallest estimate. We see that the number of localizations required to reach a stable resolution estimate depends on the structure. However, all the curves exhibit the same trend of an underestimated resolution that converges to a plateau once a certain number of localizations are exceeded.

Figure 2m shows the same curves as Fig. 2l but with the number of localizations normalized to the area covered by the structure. The sample area is estimated directly from the image rendered at the highest localization density with a pixel size of 5 nm and estimated as the number of pixels with a value greater than 0.5 multiplied by the area of a single pixel. We see from the proposed normalization that the five images have similar localization density as well as a relatively similar threshold for the convergence of the resolution estimate of about 1–4 × 10^4^ loc. per µm^2^.

We note that the estimated sample area and, therefore the localization density can strongly vary depending on the chosen pixel size and threshold (5 nm and 0.5 in this manuscript; both values were found, based on visual inspection, to produce an adequate estimate of the sample area for all the datasets presented). To be able to compare the localization density from different experiments, it is mandatory to use the same pixel size and threshold.

### Experimental results: DNA-PAINT

We also applied our method to DNA-PAINT data of microtubules in COS-7 cells (courtesy of F. Schueder and R. Jungmann, reported localization precision of ~5.5 nm^12^). The dataset considered has a reported localization uncertainty of about 8 nm.

Figure 3a–c show the bilinear histogram rendering of three randomly selected fields-of-view. Figure 3d shows the estimated resolution as a function of the pixel size. Figure 3e shows the estimated resolution as a function of the localization density. For each sample point, the resolution is estimated as a function of the pixel size and the smallest resolution is retained. We observe that a localization density smaller than about 10^4^ loc. per µm^2^ is not sufficient for our method to output a resolution estimate. Past this threshold, we see that the resolution gradually improves with the localization density and stabilizes at around 3–4×10^4^ loc. per µm^2^. These numbers are consistent with the results obtained with the shareloc data. Finally, Fig. 3f shows a zoom-in of Fig. 3c. The ability to visually resolve the microtubule hollowness confirms the high resolution predicted by our algorithm.

### Experimental results: Multi-color STORM

**Fig. 4:**
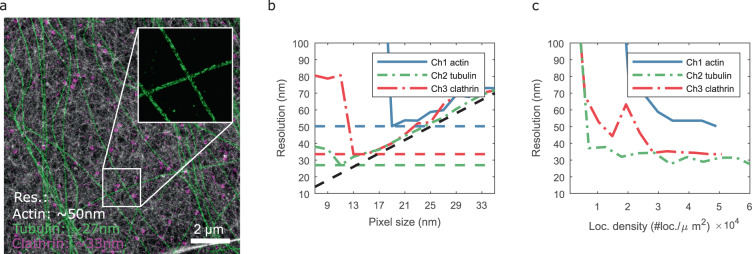
Resolution estimation of multi-color STORM data. **a** Bilinear histogram rendering at 5 nm pixel size. Actin (phalloidin-AF647; STORM; gray colormap), microtubules (two anti-α-tubulin antibodies; STORM; green colormap) and clathrin-coated pits (anti-clathrin light chain; Atto655 imager; DNA-PAINT; red colormap). **b** Resolution vs pixel size. **c** Resolution as a function of the loc. density. Scale bar: 2 µm.

Finally, we applied our method to multi-color STORM/DNA-PAINT data (courtesy of A. Jimenez and C. Leterrier, reported localization precision of ~4 nm, Fig. 6a of ref. ^13^). Bilinear histogram rendering of a 12 × 12 µm^2^ selected ROI is shown in Fig. 4a. We show in the inset of Fig. 4a, a zoom-in of the tubulin channel where the microtubule hollowness is also clearly visible.

Figure 4b shows the resolution as a function of the pixel size for all the channels. Figure 4c shows the resolution estimate as a function of the localization density. Again, we observe that a localization density of about 10^4^ loc./µm^2^ is required to get a first estimate. As we include more localizations, the resolution gradually improves until it reaches a plateau at about 2–4 × 10^4^ loc. per µm^2^.

## Discussion

As previously shown^1^, localization-uncertainty-based Gaussian rendering can also provide a reasonable resolution estimate, but is making additional assumptions about the localization statistics and requires correct estimation of the individual localization uncertainty^1^. On the other hand, using a constant Gaussian kernel can bias the resolution estimate and should not be used with decorrelation analysis^6^. Bilinear histogram rendering is the method of choice since it uses the smallest amount of information about the localizations, their position. It also alleviates the rendering rounding error compared to standard histogram rendering, thus requiring a significantly lower number of localizations enabling reliable resolution estimation for state of the art SMLM data.

Here, we have shown how the resolution is a function of the rendering pixel size for bilinear histogram rendering. To find the optimal pixel size (balance between sampling and density), we advise to compute the resolution as a function of the pixel size and retain the smallest resolution achieved. To assess the labeling density, we computed the resolution as a function of the number of localizations included in the rendering. Since the optimal pixel size depends on the number of localizations included, we advise to again compute the resolution as a function of the pixel size and retain the smallest resolution achieved. The presence of a plateau is a reliable indicator of sufficient labeling density. The average runtime for the estimation of the resolution of a single experimental dataset over a field-of-view of 12 × 12 µm is about 30 seconds (see Supplementary Information, Note 4). Using a variety of experimental datasets, we have shown that a localization density of about 1–4 × 10^4^ loc. per µm^2^ was required for the resolution estimation to converge. A Matlab implementation of the bilinear histogram method used in this work as well as a basic script for processing of localization data is publicly available at https://github.com/Ades91/ImDecorr^12^.

## Methods

The localization dataset is loaded into Matlab. The x and y localization positions are converted to nanometers. All the localizations outside of the specified field-of-view are then filtered. To estimate the localization density, we have to estimate the sample surface. To estimate the sample surface, we render all the localizations using bilinear histogram rendering with a pixel size of 5 nm. The resulting image is then binarized with a threshold of 0.5. The surface is given as the number of pixels with a value >0.5 times the area of a single pixel. The localization density is then given by the number of localizations included in the rendering divided by the sample area. Then for each localization density and each pixel size, we render the localization and estimate the image resolution using decorrelation analysis^1^.

### Reporting summary

Further information on research design is available in the Nature Research Reporting Summary linked to this article.

## Supplementary information

Supplementary Information

Description of Additional Supplementary Files

Supplementary Data 1

Reporting Summary

## Data Availability

The shareloc data are publicly available at https://shareloc.xyz/#/. Data requests for DNA paint datasets must be addressed to Florian Shueder and Ralf Jungmann. Datasets for multi-color STORM are publicly available https://figshare.com/articles/dataset/Source_Data_for_Figure_6_of_Jimenez_et_al_Methods_2020/12279917.

## Code availability

All the codes used in this manuscript are publicly available at https://github.com/Ades91/ImDecorr.
