# Supplementary material for: Parameter-free rendering of single-molecule localization microscopy data for parameter-free resolution estimation
Source: Commun Biol. 2021 May 11;4:550. doi: 10.1038/s42003-021-02086-1 (PMC8113488; doi:10.1038/s42003-021-02086-1)
Supplement: Supplementary file 1 — Supplementary Information [file 42003_2021_2086_MOESM1_ESM.pdf]

Supplementary Information: Parameter-free rendering of Single-Molecule Localization Microscopy data for parameter-free resolution estimation

Adrien C. Descloux[1], *Kristin S. Grußmayer*[1,2], *Aleksandra Radenovic*[1]

[1]*École Polytechnique Fédérale de Lausanne, Laboratory of Nanoscale Biology, 1015 Lausanne, Switzerland*

[2]*Present address : Delft University of Technology, Grußmayer Lab, Department of Bionanoscience, Kavli Institute of Nanoscience, 2628HZ Delft, The Netherlands*

Supplementary note 1: Standard and Bilinear histogram rendering, ShareLoc dataset selection

Supplementary figure 1 illustrates the difference between bilinear and standard histogram rendering. Supplementary figure 1a, c and e shows a 3 microns field-of-view rendering of Fig. 1 ShareLoc (https://shareloc.xyz/#/) datasets using both bilinear histogram rendering (top left) and standard histogram rendering (bottom right). A trained eye can see that the standard histogram rendering looks more pixelated while bilinear histogram image appears slightly smoother. Based solely on the visual impression, it seems that both images look similar enough and that it should not matter for further resolution estimation.

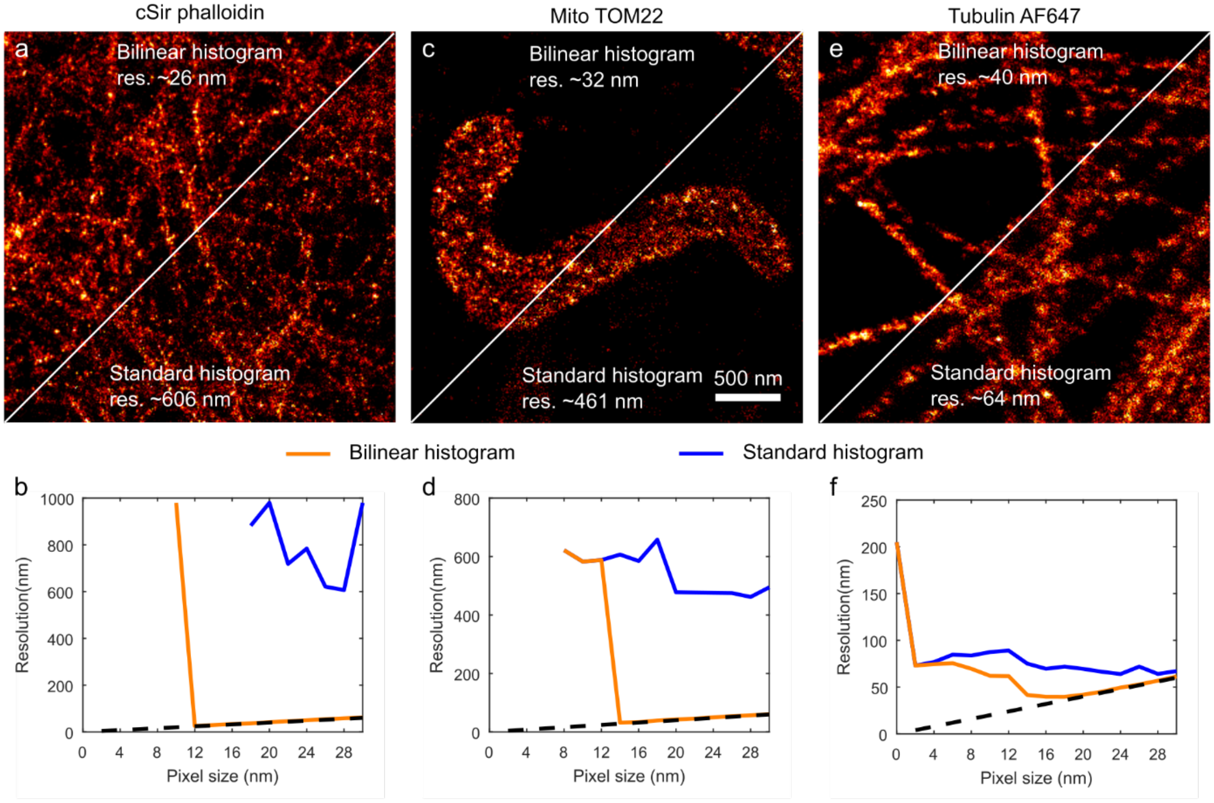

Supplementary figure 1 Bilinear histogram rendering vs standard histogram rendering. (a) cSir phalloidin (c) Mitochondria TIM22 (e) Tubulin AF646. (b-d-f) Estimated resolution as a function of the rendering pixel size of (a-c-e) correspondingly. Field of view 3 µm, Scale bar 500 nm

Supplementary figure 1b, d and f show the estimated resolution as a function of the pixel size for both rendering methods. Despite the visual similarity between the two images, the resolution estimate varies dramatically, with the standard histogram rendering producing completely underestimated resolution. The reason why we show only three datasets instead of the five shown in Fig. 1 is that no resolution estimate (i.e. curves without any local maxima) could be obtained for WGA and gp210 datasets for standard histogram rendering.

Supplementary Note 2: Gaussian rendering, ShareLoc dataset selection

Supplementary figure 2a shows the rendering of the same dataset (cSIT Phalloidin, see Fig. 2 for more details) using bilinear histogram or Gaussian rendering (Kernel size of 15 nm, pixel size of 15 nm for both; FOV of 3x3 µm). Supplementary figure 2b and c shows the zoom-in of the dashed white rectangle indicated Supplementary figure 2a. Supplementary figure 2d shows how the estimated resolution changes as a function of the Kernel size used. As expected, we see that the estimated resolution is evolving almost linearly with the size of the Gaussian Kernel. As it is already discussed in greater details in [1], the Gaussian kernel is biasing the resolution estimate by filtering the Fourier space of the image, creating artificial local maxima in the decorrelation analysis. We repeated the same calculation for two other datasets (Supplementary figure 2e and f, Mito TOM22 and Tubulin AF647 respectively), and found a similar behavior.

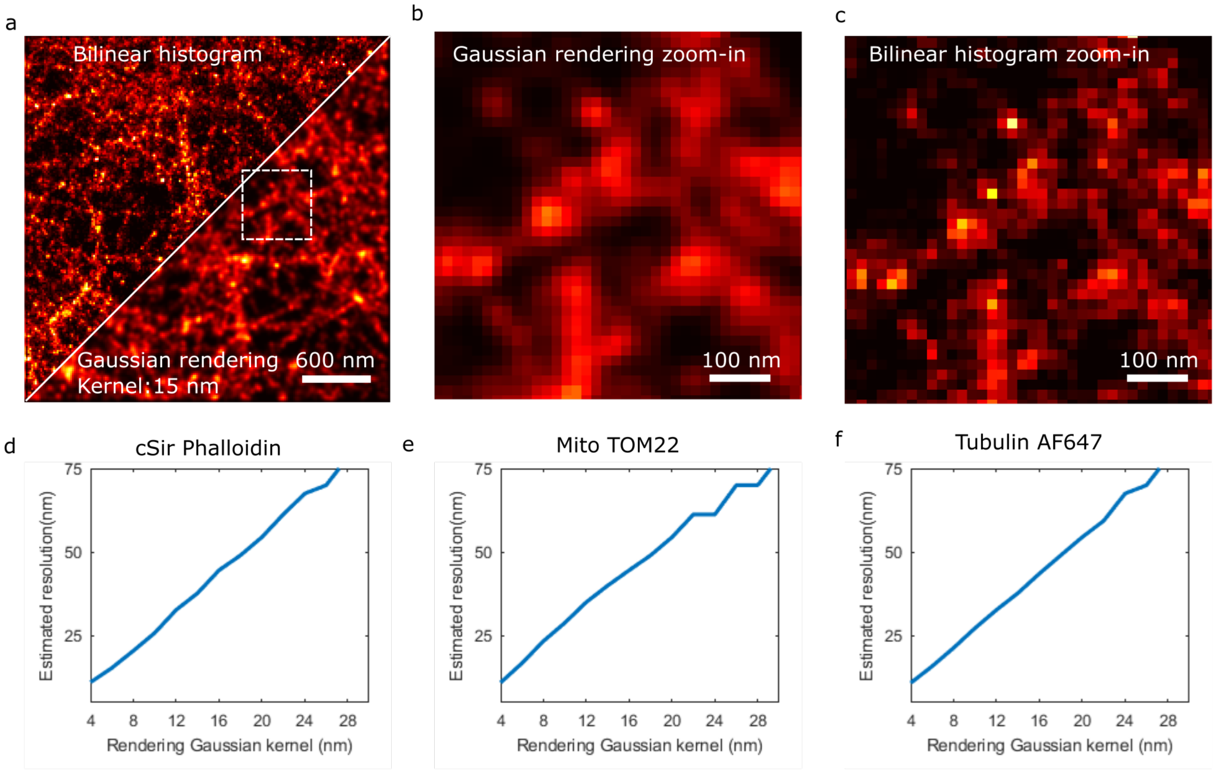

Supplementary figure 2 Comparison of Gaussian rendering with bilinear histogram rendering. (a) Rendering of the central part of cSIr phalloidin dataset (FOV of 3x3 µm; scale bar 600 nm) using bilinear and Gaussian rendering (pixel size and Gaussian kernel size of 15 nm). (b) and (c) Zoom-in of the 600x600 nm ROI indicated in (a) by the dashed white rectangle for Gaussian and bilinear histogram rendering; scale bar 100 nm. (d),(e) and (f) Estimated resolution as a function of the rendering Gaussian kernel for 3 datasets, cSir Phalloidin, Mito TOM22 and Tubulin AF647 respectively.

These results suggest that fixed Gaussian rendering should not be used when assessing the resolution based on decorrelation analysis.

Supplementary note 3: Simulations of Bilinear and Standard histogram rendering

To further investigate the difference between bilinear and standard histogram rendering, we performed simulations of lines with decreasing spacing (30 to 2 nm in steps of 2 nm, line width of 2 nm; see Supplementary figure 3a, pixel size of 5 nm, 10^4 localizations). To restrict the discussion to the question of rendering, we directly simulate the localization procedure and do not discuss how different SMLM analysis software might influence the results. A detailed comparison of the performance of localization software can be found in [2,3] and it is the task of the user to select the appropriate software.
For each localization the x-y position is computed by randomly selecting an emitter, and adding to its ground-truth location a normally distributed offset with a set standard deviation σ_{gt}. The result of the simulation is a list of positions that stochastically sample the underlying structure with a certain accuracy.

We performed the simulations for 3 and 6 nm localization precision, which are comparable with values reported for the experimental datasets processed in this work. Supplementary figure 3b shows the resolution evolution as a function of the number of localizations included in the rendering. For each number of localization included in the rendering, we computed the resolution as a function of the pixel size and retained the smallest estimate. We see that both rendering methods (solid curves: bilinear histogram, dashed curves, standard histogram) are forming a plateau while reaching the theoretical resolution (dashed black lines). However, standard histogram rendering requires a significantly larger number of localizations (please note the logarithmic scale) to compensate for the rounding error.

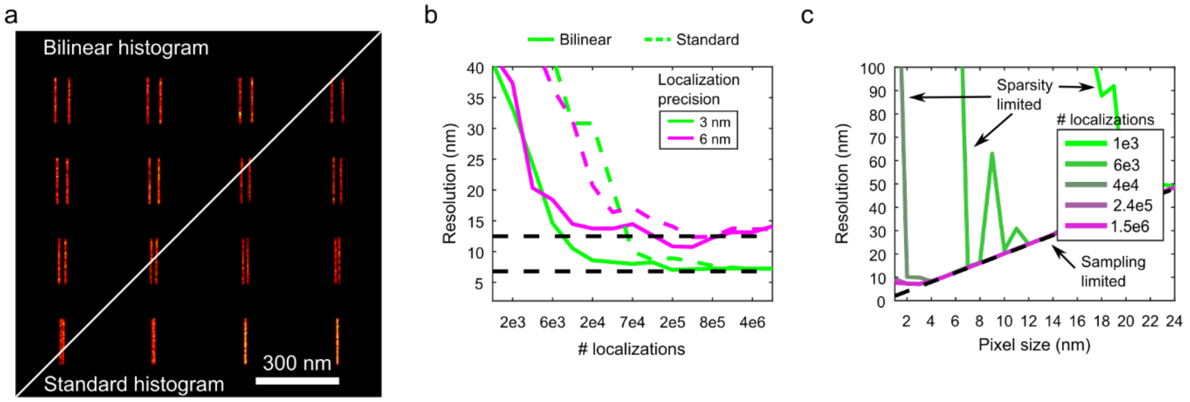

Supplementary figure 3 Bilinear and standard histogram rendering simulations. (a) Bilinear and standard histogram rendering of simulated localizations; pixel size of 5 nm, 10^4 localizations. (b) Resolution as a function of the number of localizations in logarithmic scale for 3 and 6 nm input localization precision (Solid curves: bilinear histogram; Dashed colored curves: standard histogram; Dashed black lines: Theoretical resolution from input localization precision) (c) Resolution as a function of the bilinear histogram pixel size for several number of localizations (σ_{gt}=3

nm). Scale bar: 300 nm.

We show in Supplementary figure 3c the influence of the choice of the pixel size over the resolution estimate (bilinear histogram, σ_{gt}=3nm). We see three distinct regimes. For too large pixel size, the image is sampling limited and the resolution increases linearly with the pixel size. If the pixel size is too small, the localizations are spread and do not correlate spatially. Finally, if the number of localizations is too low, the resolution estimate will not reach its theoretical minimum.

Supplementary note 4: Bilinear rendering and resolution estimate runtime

Since the methodology we propose to estimate the resolution of SMLM datasets relies on the iterative processing of the input data, we performed runtime assessments of the rendering and the resolution estimate. All the results shown were obtained using a Windows 10 installation of Matlab (R2017b), an Intel i7-3960X CPU @ 3.3GHz and an NVIDIA GeForce GTX 480 (code available https://github.com/Ades91/ImDecorr).

Supplementary figure 4a shows the average time required to render a randomly generated localization dataset as a function of the image side length (given by the image field-of-view divided by the chosen rendering pixel size) for several numbers of localizations included in the rendering. We see that even in the extreme case of a 12’000 pixels x12’000 pixels image and 10^7 localizations, the rendering time barely exceeds 3 seconds and increases linearly with the image side length Supplementary figure 4b shows the same data plotted as a function of the number of localizations, showing that the rendering of bilinear histogram time increases linearly with the number of localizations.

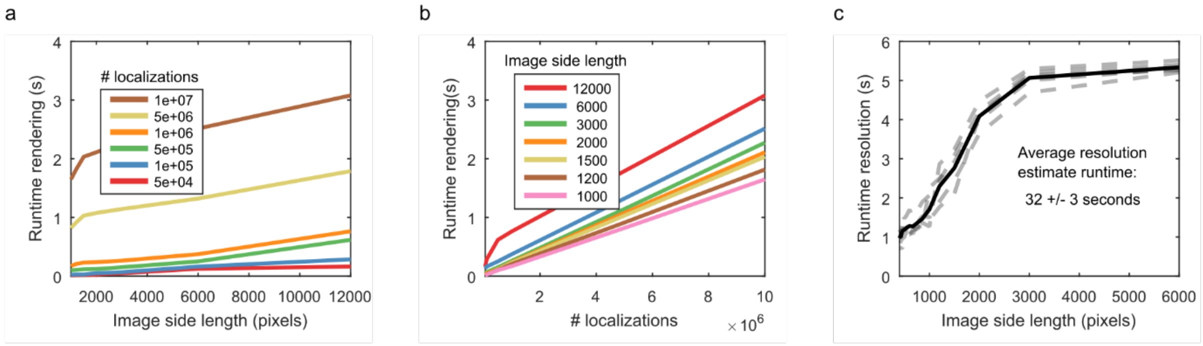

Supplementary figure 4 Rendering and resolution estimate runtime. (a) Bilinear rendering runtime as a function of the rendered image side length for several number of localizations included in the rendering. (b) Same data as (a) plotted as a function of the number of localizations for several rendered image side lengths. (c) Resolution estimate runtime (rendering and resolution estimate) as a function of the rendered image size for each 5 shareLoc datasets (dashed light gray curves). The black curve indicates the average runtime. The total processing time is given by the sum of runtime for each image side length and is about 32±3 seconds.

Finally, the Supplementary figure 4c shows the runtime for the full processing pipeline (rendering and resolution estimation) as a function of the rendered image side length. We used the 5 shareLoc datasets as input data, using all the localizations available. We rendered a field-of-view of 12 µm x12 µm at pixel sizes ranging from 2 to 30 nm, resulting in the image side length of 12’000/30=400 pixels to

12’000/2=6’000 pixels. The light gray dashed lines corresponds to the runtime (rendering + resolution estimation) of the 5 datasets as a function of the image side length (proportional to the inverse of the pixel size). The solid line corresponds to the average runtime. Since the resolution estimation procedure requires to loop over all the pixel sizes, the total time required to get a final resolution estimate is given by the sum of runtime for each image side length. We found an average computation time of 32 ± 3 seconds (N = 5), which we believe to be representative of the minimal time required to estimate the resolution of a localization microscopy dataset using our methodology.

Supplementary references

[1] Descloux, A., Grußmayer, K. S., & Radenovic, A. (2020). Addendum: Parameter-free image resolution estimation based on decorrelation analysis. Nature Methods, 17(10), 1061-1063.
