# Supplementary material for: Parameter-free rendering of single-molecule localization microscopy data for parameter-free resolution estimation
Source: Commun Biol. 2021 May 11;4:550. doi: 10.1038/s42003-021-02086-1 (PMC8113488; doi:10.1038/s42003-021-02086-1)
Supplement: Supplementary file 2 — Description of Additional Supplementary Files [file 42003_2021_2086_MOESM2_ESM.pdf]

Description of Additional Supplementary Files

File name: Supplementary Data 1
Description: Data used to plot graphs in all Figures.
